# Research progress on the mechanism, diagnosis, and treatment of exosomal miRNA in chronic prostatitis

**DOI:** 10.3389/fimmu.2026.1888573

**Published:** 2026-07-22

**Authors:** Yihang Shen, Baojun Ju

**Affiliations:** 1 Department of Andrology, The First Affiliated Hospital of Henan University of Chinese Medicine, Zhengzhou, Henan, China; 2 The First Clinical Medical College of Henan University of Chinese Medicine, Zhengzhou, Henan, China

**Keywords:** chronic prostatitis, CP/CPPS, diagnosis, exosomes, microRNA (miRNA), research progress

## Abstract

Chronic prostatitis/chronic pelvic pain syndrome (CP/CPPS) is a common condition that significantly impairs men’s quality of life. Its pathogenesis remains incompletely understood, and there is a lack of specific diagnostic methods. As important signaling molecules in intercellular communication, microRNAs (miRNAs) in exosomes play a key role in the pathological progression of CP/CPPS. This review proposes that ‘exosome miRNA-mediated interactions within the local urogenital microenvironment’ constitute the core mechanism driving the chronicity and localization of CP/CPPS. We provide a comprehensive overview of the diagnostic and prognostic value of specific exosome miRNA expression profiles detected in the biofluids (such as prostatic fluid and urine) of CP/CPPS patients. Furthermore, we explore the dual role that exosomal miRNAs play in disease initiation and progression by regulating inflammation, oxidative stress, and immune responses. Finally, we discuss the potential of exosome-based miRNA delivery systems as novel targeted therapeutic strategies for CP/CPPS. In summary, exosomal miRNAs are of great significance for understanding the complex pathological networks underlying CP/CPPS. Future research should focus on the validation of specific biomarkers, the elucidation of intercellular communication networks, and the advancement of their clinical translation and application.

## 1 Introduction

Chronic prostatitis/chronic pelvic pain syndrome (CP/CPPS) is primarily characterized by urinary dysfunction, pelvic floor pain, and sexual dysfunction. It affects up to 50% of men at some point in their lives and significantly impairs their quality of life (1, 2). Its pathogenesis involves inflammation, immune dysregulation, and oxidative stress (3). Clinical treatment often includes anti-inflammatory drugs, α-blockers, physical therapy, and anxiolytics or antidepressants. However, these approaches may cause adverse effects, such as gastrointestinal reactions, and can lead to poor patient compliance (4). Consequently, there is an urgent need to explore novel diagnostic and therapeutic strategies with high specificity and minimal invasiveness.

Exosomes are nanoscale vesicles secreted by cells that carry a cargo of bioactive molecules, including nucleic acids, lipids, and proteins. They are implicated in various physiological and pathological processes, such as immunity (5), neural transmission (6), reproduction (7), and inflammation (8). Among these, exosomal microRNAs (miRNAs) have emerged as key regulators of chronic inflammation (9). By synthesizing available evidence, this review proposes a core hypothesis of an “exosomal miRNA-mediated local microenvironment crosstalk mechanism.” This narrative review synthesizes the current literature on exosomal miRNAs in CP/CPPS by summarizing key findings from original research articles, clinical studies, and preclinical models. Literature was identified through searches of PubMed, Web of Science, and Scopus databases using combinations of keywords including ‘exosomes,’ ‘microRNA,’ ‘chronic prostatitis,’ ‘CP/CPPS,’ ‘biomarker,’ and ‘therapy.’ Priority was given to studies published in English that directly investigated exosomal or extracellular vesicle-associated miRNAs in CP/CPPS contexts. While we aimed for comprehensive coverage, this is a narrative review and does not claim to be a formal systematic review with meta-analysis.

## 2 Characteristics and functions of exosomal miRNAs

Exosomes are a subclass of extracellular vesicles (EVs) with diameters ranging from 30 to 150 nm. Enclosed by a phospholipid bilayer, they harbor cytoplasmic constituents and function as pivotal mediators of intercellular communication (10, 11).Their biogenesis initiates with the inward budding of endosomal membranes to form early endosomes, which mature into multivesicular bodies (MVBs) containing intraluminal vesicles. Upon fusion of MVBs with the plasma membrane, these vesicles are released into the extracellular space as exosomes (12). Ubiquitously distributed in various biological fluids, exosomes carry a diverse cargo comprising nucleic acids, proteins, lipids, and metabolites. Among these, miRNAs act as critical signaling molecules. Encapsulated within exosomes, they specifically bind to recipient cells via surface proteins and modulate gene expression in target cells (13).

MiRNAs are short, non-coding RNA molecules, typically 19–25 nucleotides in length. A single miRNA can target hundreds of messenger RNAs (mRNAs), thereby regulating gene expression at the post-transcriptional level. They are ubiquitously present in cells, tissues, and body fluids and are closely associated with diverse physiological and pathological processes, including metabolism, immunity, inflammation, and tumorigenesis (14, 15). Mature miRNAs exert their functions by binding to Argonaute (Ago) proteins to form the miRNA-induced silencing complex (miRISC). Through complementary base pairing between the “seed sequence” (typically nucleotides 2–8 of the miRNA) and the 3’-untranslated region (3’-UTR) of target mRNAs, this complex either represses mRNA translation or promotes mRNA degradation (16).

The packaging of miRNAs into exosomes is not a random process but a highly selective one. MVBs, the precursors of exosomes, are closely associated with processing bodies (P-bodies) in the cytoplasm. P-bodies are sites for post-transcriptional mRNA regulation and are enriched with core components of the RNA-induced silencing complex (RISC), such as AGO2 and GW182 proteins. This spatial association suggests that the incorporation of miRNAs into exosomes is intimately linked to their functional silencing complexes (17). Upon internalization by recipient cells, the miRISC complexes carried by exosomes induce post-transcriptional silencing of target mRNAs, thereby modulating the function of recipient cells (18). Consequently, exosomal miRNAs are considered an efficient, long-distance “language” for intercellular communication, playing a vital role in coordinating cellular responses (19). The selective loading and targeted delivery of exosomal miRNAs establish them as key messenger molecules in the context of CP/CPPS.

Notably, the selective loading and targeted delivery of exosomal miRNAs position them as significant players in inflammation-related diseases. In the context of CP/CPPS—a condition characterized by local inflammation and immune dysregulation—complex communication networks involving multiple immune and stromal cells are well documented (20). As key intercellular signaling molecules, exosomal miRNAs may contribute to the pathogenesis and progression of CP/CPPS by modulating inflammation-related gene expression in target cells. Consequently, in-depth investigation into the altered expression profiles and functional mechanisms of exosomal miRNAs in CP/CPPS could elucidate their role in the disease’s underlying pathology and provide a theoretical foundation for identifying novel diagnostic biomarkers and therapeutic targets.

## 3 Characteristics of exosomal miRNA expression profiles in CP/CPPS patients

### 3.1 Characteristics of expression profiles in expressed prostatic secretions

Expressed prostatic secretion (EPS) represents the most direct biological fluid for reflecting the local prostatic microenvironment. In CP/CPPS patients, the expression profiles of exosomal miRNAs in EPS of CP/CPPS are significantly altered. Chen et al. (21) performed high-throughput sequencing on EPS samples from CP/CPPS patients and healthy controls, identifying 22 highly abundant miRNAs that were significantly upregulated (≥2-fold). Among these, miR-21-5p, miR-30a-5p, miR-30d-5p, miR-103a-3p, and miR-141-3p exhibited the most pronounced differences. Subsequent verification by quantitative real-time PCR (qRT-PCR) confirmed the consistent upregulation of these five miRNAs in the EPS of CP/CPPS patients, thereby establishing the reliability of this expression profile. Notably, follow-up analysis revealed that alleviation of pelvic pain symptoms was accompanied by a significant decrease in the levels of miR-21-5p, miR-103a-3p, and miR-141-3p, underscoring their potential as integrated biomarkers for both diagnosis and prognosis.

In a separate study, Ouyang et al. (22) profiled miRNAs in EPS from CP/CPPS patients and healthy individuals and identified a panel of differentially expressed pain-related miRNAs, including miR-204-5p, let-7d-3p, let-7b-3p, let-7c-3p, miR-146a-5p, and miR-320a-5p. This expression signature holds promise as a specific diagnostic biomarker for CP/CPPS. Furctional analysis further revealed that miR-21-5p is a classic pro-inflammatory factor (23), whereas members of the let-7 family and miR-146a-5p are involved in the negative feedback regulation of inflammation (24, 25). These findings suggests that the exosomal miRNA profile in the EPS of CP/CPPS patients constitutes a functionally coordinated network-an integrated modular whole-rather than mere collection of isolated molecules. Therefore, future diagnostic strategies should shift from the pursuit of a single “gold standard” biomarker toward the construction of diagnostic models based on multi-gene functional network.

### 3.2 Characteristics of expression profiles in urine

Urine is a readily accessible biofluid, and urinary exosomal miRNAs have emerged as promising biomarkers for early disease detection (26). Schneider et al. (27) identified a panel of eight prostate cancer-associated miRNAs—including hsa-miR-501, hsa-miR-20a, and hsa-miR-106—that were significantly upregulated in urinary exosomes from patients with CP/CPPS. In subsequent functional assays, co-culture of the human monocyte cell line THP-1 with these patient-derived urinary exosomes demonstrated efficient cellular internalization. Notably, this internalization maintained THP-1 cells in an M0-like state, inhibited M2 polarization, and prevented their transition to an anti-inflammatory phenotype, thereby disrupting immune homeostasis. This study not only validates urinary exosomal miRNAs as a non-invasive diagnostic resource for CP/CPPS but also suggests a potential molecular link between CP/CPPS and prostate carcinogenesis, given the significant overlap between these miRNAs and established prostate cancer biomarkers. These findings provide a strong rationale for long-term follow-up and risk stratification in patients with CP/CPPS.

### 3.3 Characteristics of expression profiles in blood

Blood represents another critical biofluid for investigation. However, in contrast to EPS and urine, exosomal miRNAs in the circulation of patients with CP/CPPS lack a distinctive expression signature, showing no significant deviations in molecular composition compared to healthy controls. Consequently, blood-derived exosomal miRNAs appear unsuitable as biomarkers for distinguishing CP/CPPS patients from healthy individuals. Functionally, circulating exosomes from CP/CPPS patients fail to induce systemic inflammation (27). This absence of systemic signatures suggests that CP/CPPS-associated molecular alterations are confined locally to the genitourinary tract (reflected in EPS and urine). Upon entering the bloodstream, these disease-specific signals may become diluted below detection limits or remain absent, reinforcing the characterization of CP/CPPS as a localized disorder. These observations offer crucial guidance for clinical biomarker development, advocating for the prioritization of EPS and urine to maximize both diagnostic specificity and practical utility. The expression of miRNAs in body fluids is summarized in **Table 1**.

**Table 1:** Characteristics of exosomal miRNA expression profiles in body fluids of CP/CPPS patients.

Body fluid source	Differentially expressed miRNAs	Expression change	Characteristics and diagnostic potential	Reference
Expressed Prostatic Secretions (EPS)	miR-21-5p, miR-30a-5p, miR-30d-5p, miR-103a-3p, miR-141-3p	Significantly Upregulated	miR-21-5p shows the highest diagnostic accuracy; levels of miR-21-5p, miR-103a-3p, and miR-141-3p decrease with symptom alleviation, with potential for both diagnosis and prognosis.	(21)
Expressed Prostatic Secretions (EPS)	miR-204-5p, let-7d-3p, let-7b-3p, let-7c-3p, miR-146a-5p, miR-320a-5p	Differentially Expressed	Associated with pain, showing great potential as a specific diagnostic biomarker for CP/CPPS.	(22)
Urine	hsa-miR-501, hsa-miR-20a, hsa-miR-106, etc. (total 8)	Significantly Upregulated	These are prostate cancer-specific miRNAs; functionally, they can be taken up by cells, inhibit the transition of immune cells to an anti-inflammatory state, and disrupt immune balance; suggesting a potential association between CP/CPPS and prostate cancer risk.	(27)
Blood	No characteristic expression profile identified	No Significant Difference	No significant difference in molecular composition compared to healthy individuals; cannot be used as a biomarker to distinguish CP/CPPS.	(27)

## 4 Directly validated mechanisms of exosomal miRNAs in CP/CPPS pathogenesis

Building on the differentially expressed exosomal miRNAs identified in patients with CP/CPPS (detailed in Section 3), this section dissects their functional roles within the conceptual framework of ‘local microenvironment crosstalk.’ These miRNAs do not operate in isolation; rather, they collectively orchestrate the pathogenesis and chronification of CP/CPPS by regulating an intricate network of pathological processes. Key mechanisms validated in CP/CPPS models include the initiation and amplification of pro-inflammatory cascades, the disruption of anti-inflammatory homeostasis, the induction of oxidative stress, and the modulation of immune cell functions. Importantly, these pathways do not function independently but engage in dynamic crosstalk, forming self-perpetuating positive feedback loops that exacerbate the disease state. For instance, pro-inflammatory mediators can trigger oxidative stress, thereby fueling further inflammation, while impaired anti-inflammatory regulation may hinder inflammatory resolution and promote disease persistence. By elucidating how exosomal miRNAs coordinate these multifaceted and interconnected pathways, this section aims to provide a comprehensive understanding of their role in shaping the prostatic niche and to highlight critical nodes for therapeutic intervention. Mechanisms discussed in this section are limited to those directly validated in CP/CPPS models; putative mechanisms inferred from other disease models are presented separately in Section 5 as testable hypotheses for future research.

### 4.1 Pro-inflammatory mechanisms

#### 4.1.1 miR-155 mediates prostatic inflammation via the TLR4/NF-κB axis

The pro-inflammatory role of exosomal miR-155 in CP/CPPS pathogenesis has been established through both patient-derived exosome studies and genetic loss-of-function approaches.

Zhao et al. (28) demonstrated that exosomes are abundantly enriched in the prostatic fluid of CP/CPPS-A patients and selectively overloaded with miR-155. These exosomes are efficiently internalized by prostate stromal cells and activate IL-8 and TNF-α expression *in vitro*, with exosomal membrane integrity serving as a prerequisite for this signaling effect. *In vivo*, intraprostatic injection of patient-derived exosomes induces prostatitis in rats, accompanied by excessive activation of IL-8, TNF-α, and iNOS (28). These findings position prostate fluid exosomes and their miR-155 cargo as active participants in the inflammatory cascade of CP/CPPS-A.

The causal significance of miR-155 has been further corroborated through loss-of-function studies in the EAP model (29). miR-155 knockout mice exhibit attenuated pelvic tactile allodynia/hyperalgesia and suppressed TLR4/NF-κB pathway activation compared to wild-type controls. Mechanistically, LPS administration—acting as a TLR4 ligand—enhances miR-155 upregulation and TLR4/NF-κB activation in prostatic tissues of wild-type EAP mice, while miR-155 deficiency reverses this effect. The inflammatory and oxidative stress profiles in prostatitis mice are characterized by elevated IL-1β, TNF-α, IL-6, IFN-γ, IL-12, and MDA, alongside reduced IL-10, SOD, and GSH-Px, with unaltered IL-4 levels (29). Collectively, these findings indicate that miR-155 deficiency ameliorates pelvic pain and improves both inflammation and oxidative stress in prostatic tissues through TLR4-dependent NF-κB modulation.

#### 4.1.2 Epithelial-derived miR-203a-3p facilitates stromal inflammation via the DUSP5/ERK1/2/MCP-1 axis

In addition to the miR-155 pathway, a distinct mechanism of epithelial-stromal crosstalk mediated by exosomal miR-203a-3p has been identified in CP/CPPS-A (30). Inflammatory prostate epithelial cells release miR-203a-3p-rich exosomes, which are taken up by prostate stromal cells and promote stromal inflammation through upregulation of MCP-1. Mechanistically, DUSP5 is a direct target gene of miR-203a-3p, and its suppression leads to activation of the ERK1/2/MCP-1 signaling pathway (30). The pro-inflammatory effects of exosomes derived from CP/CPPS-A patient prostatic fluids are consistent with those of exosomes derived from inflamed epithelial cells, confirming the clinical relevance of this pathway.

Notably, this miR-203a-3p-mediated pathway operates through a different cellular origin than miR-155: while miR-155 is predominantly elevated in prostate stromal cells (28), miR-203a-3p is secreted by epithelial cells and acts upon stromal cells (30). This cell-type-specific signaling exemplifies the complexity of exosomal miRNA-mediated intercellular communication within the prostatic microenvironment.

Importantly, therapeutic intervention targeting this pathway has demonstrated preclinical efficacy: miR-203a-3p antagomir-loaded exosomes derived from prostate epithelial cells successfully target the prostate and alleviate prostatitis by inhibiting the DUSP5-ERK1/2 pathway (30), offering a promising strategy for CP/CPPS-A treatment.

#### 4.1.3 Integrated perspective on pro-inflammatory miRNA networks

Collectively, the evidence discussed above establishes two distinct but complementary pro-inflammatory axes mediated by exosomal miRNAs in CP/CPPS: (1) the miR-155/TLR4/NF-κB pathway, validated through both patient-derived exosome studies and genetic knockout models; and (2) the epithelial-derived miR-203a-3p/DUSP5/ERK1/2/MCP-1 pathway, which orchestrates stromal inflammation through cell-type-specific crosstalk. These pathways do not operate in isolation. The inflammatory cytokines released through these mechanisms—including TNF-α, IL-1β, and IL-6—can directly induce oxidative stress, as evidenced by elevated MDA and reduced SOD/GSH-Px levels in prostatitis models (29). This reciprocal reinforcement between inflammation and oxidative stress establishes a self-perpetuating vicious cycle that drives disease chronicity and progression in CP/CPPS.

### 4.2 miRNA-mediated anti-inflammatory mechanisms

In contrast to the pro-inflammatory pathways driven by miR-155 and miR-203a-3p, miR-146a-5p functions as a critical brake on TLR4/NF-κB signaling, and its dysregulation in the prostatic microenvironment contributes to the loss of inflammatory homeostasis in CP/CPPS.

Lu et al. (31) investigated the role of prostasome-derived miR-146a in regulating the TLR-4/NF-κB pathway in experimental autoimmune prostatitis (EAP) rats and evaluated the therapeutic effects of Anemone tomentosa. The authors found that miR-146a-5p expression in prostasomes was significantly downregulated in EAP model rats compared with normal controls. Treatment with medium- and high-dose Anemone tomentosa as well as miR-146a mimics significantly upregulated prostasomal miR-146a-5p expression, while miR-146a inhibitor further suppressed its expression.

Mechanistically, miR-146a-5p negatively regulates TLR-4/NF-κB signaling by targeting TRAF6, a key adaptor molecule in the TLR signaling pathway. Both Anemone tomentosa and miR-146a mimics reduced the protein expression of TLR-4, p-IκBα, and TRAF6 in prostate tissues while promoting IκBα upregulation, thereby inhibiting NF-κB activation. Consequently, serum levels of pro-inflammatory cytokines—including TNF-α, IL-6, IL-8, and IFN-γ—were significantly decreased. Histological examination confirmed that Anemone tomentosa and miR-146a mimics attenuated inflammatory cell infiltration and ameliorated pathological injury in prostate tissue, whereas miR-146a inhibitor exacerbated tissue damage (31).

These findings establish that prostasome-derived miR-146a-5p functions as a negative feedback regulator of TLR-4/NF-κB signaling by targeting TRAF6, thereby restraining excessive inflammatory responses in the prostate. The therapeutic effect of Anemone tomentosa in EAP rats is mediated, at least in part, through upregulation of prostasomal miR-146a-5p. Combined with the miR-155-driven pro-inflammatory amplification discussed in Section 4.1, the impairment of this anti-inflammatory brake establishes a permissive environment for the perpetuation of chronic inflammation.

### 4.3 miRNA-mediated immune modulation

Beyond the epithelial-stromal crosstalk discussed in previous sections, exosomal miRNAs also directly modulate immune cell function within the prostatic microenvironment. Evidence from patient-derived exosomes and engineered therapeutic vesicles reveals that miRNA-mediated regulation of monocytes and macrophages plays a critical role in the immune imbalance characteristic of CP/CPPS.

Schneider et al. (27) investigated the functional impact of CP/CPPS-derived urinary exosomes on immune cells. Exosomes were isolated from post-prostatic-massage urine of CP/CPPS type IIIb patients and healthy men, then co-cultured with THP-1 monocytes (a human leukemia monocytic cell line). CP/CPPS-derived urine exosomes induced upregulation of PCa-associated proinflammatory genes, including CCR2 and TLR2, as well as proto-oncogene transcription factors such as MYB and JUNB^27]^. Furthermore, these exosomes carried specific microRNAs whose target genes were significantly enriched for pathways specific to carcinogenesis. This suggests that CP/CPPS exhibits molecular changes that may constitute a risk for prostate cancer (PCa) (27). These findings demonstrate that patient-derived urinary exosomes can actively reprogram monocyte gene expression toward a proinflammatory and oncogenic state, contributing to the persistence of chronic inflammation and potential malignant transformation in CP/CPPS.

While the above study reveals how patient-derived exosomes dysregulate immune cells and promote oncogenic inflammation, a complementary line of research has explored the therapeutic potential of engineered exosomes to correct this imbalance.

Peng et al. (32) developed a novel therapeutic approach targeting macrophage polarization in CP/CPPS. In this study, small extracellular vesicles (sEVs) derived from adipose-derived stem cells (ADSCs) were surface-modified with CD86 antibodies via click chemistry, generating CD86-sEVs capable of specifically targeting M1 macrophages (32). *In vitro*, CD86-sEVs significantly accelerated the polarization of M1 macrophages toward the anti-inflammatory M2 phenotype and upregulated anti-inflammatory factors. Consistently, *in vivo* experiments demonstrated that CD86-sEVs effectively targeted prostatic lesions, alleviated chronic pelvic pain, and suppressed inflammation by promoting the M1-to-M2 phenotypic shift (32). Importantly, miRNA array analysis identified a specific cargo of miRNAs within CD86-sEVs—including miR-26a, miR-147, miR-17, miR-21, miR-182, and miR-451a—that likely orchestrates these immunomodulatory effects (32).

Collectively, these studies establish that exosomal miRNAs exert potent immunomodulatory effects in CP/CPPS through two interconnected mechanisms: (1) patient-derived urinary exosomes carrying oncogenic miRNAs disrupt monocyte function and perpetuate inflammation by blocking M2 polarization; and (2) engineered ADSC-derived sEVs, loaded with a specific miRNA cargo, can reverse this imbalance by actively promoting M1-to-M2 macrophage transition. The identification of miRNAs such as miR-21—which appears in both contexts (as a pathogenic factor in EPS and as a therapeutic cargo in CD86-sEVs)—highlights the context-dependent nature of miRNA function and underscores the importance of delivery vehicle and cellular target in determining therapeutic versus pathogenic outcomes.

### 4.4 miRNA-mediated anti-oxidative stress

Oxidative stress is a well-established pathological feature of CP/CPPS, characterized by excessive reactive oxygen species production and compromised endogenous antioxidant defenses. Emerging evidence has identified miRNA-mediated regulation of antioxidant pathways as a direct mechanism counteracting oxidative damage in the prostatic microenvironment.

Wang et al. (33) investigated the effect of N-acetylcysteine (NAC) on carrageenan-induced chronic nonbacterial prostatitis (CNP) and the molecular mechanism involved. Male Sprague-Dawley rats were divided into control group, CNP model group, and CNP model group treated with NAC. NAC treatment significantly reduced eye scores, locomotion scores, inflammatory cell counts, Evans blue extravasation, and COX2 expression compared with the model group, confirming that NAC relieved carrageenan-induced CNP (33).

Mechanistically, NAC increased miR-141 expression and activated the Keap1/Nrf2 signaling pathway. Luciferase reporter assay confirmed that miR-141 mimic suppressed Keap1 activity and stimulated downstream target genes of Nrf2. Conversely, miR-141 inhibitor attenuated the protective effects of NAC on prostatitis (33). These findings demonstrate that NAC ameliorates carrageenan-induced prostatitis and prostate inflammation pain through miR-141 regulating Keap1/Nrf2 signaling.

**Figure 1:**
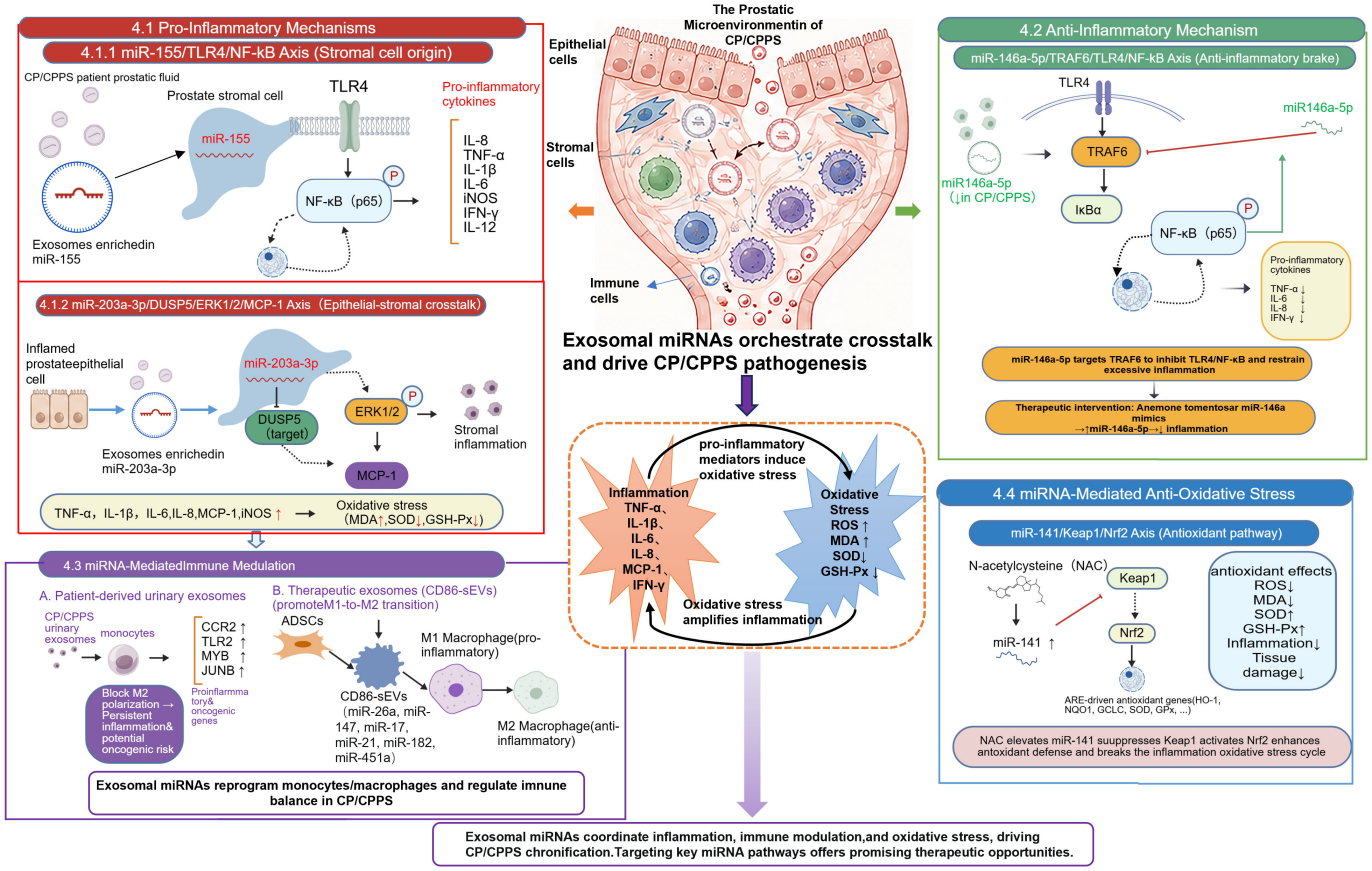
Schematic diagram of directly validated exosomal miRNA-mediated regulatory networks in CP/CPPS pathogenesis.

This study provides the first direct evidence that miRNA-mediated antioxidant regulation operates in CP/CPPS. By targeting Keap1—a negative regulator of Nrf2—miR-141 relieves Nrf2 from Keap1-mediated inhibition, allowing activation of downstream antioxidant genes. The therapeutic implication is clear: pharmacological restoration of miR-141 expression, as achieved by NAC, can break the “inflammation-oxidative stress” vicious cycle and limit tissue damage in the prostate. The specific mechanism is shown in **Table 2**. The directly validated mechanisms are summarized in **Figure 1**.

**Table 2:** The direct mechanisms of exosomal miRNA in the treatment of chronic prostatitis.

Mechanism category	miRNA	Key target/pathway	Validation status	Reference
Pro-inflammation	miR-155	TLR4/NF-κB, IL-8/TNF-α/iNOS	Validated in CP/CPPS (patient EPS + EAP KO)	(28, 29)
Pro-inflammation	miR-203a-3p	DUSP5 → ERK1/2 → MCP-1	Validated in CP/CPPS (patient EPS + *in vivo*)	(30)
Anti-inflammation	miR-146a-5p	TRAF6 → TLR4/NF-κB	Validated in CP/CPPS (EAP model)	(31)
Immune modulation	miR-501/-20a/-106	Monocyte dysfunction, M2 suppression	Validated in CP/CPPS (patient urine)	(27)
Immune modulation	miR-26a/-147/-17/-21/ -182/-451a	M1→M2 macrophage polarization	Validated in CP/CPPS (rat model)	(43)
Anti-oxidative stress	miR-141	Keap1 → Nrf2	Validated in CP/CPPS (carrageenan CNP)	(33)

## 5 Potential mechanistic hypotheses and future research directions

Beyond the directly validated mechanisms discussed in Section 4, accumulating evidence from other disease models suggests additional pathways through which exosomal miRNAs might contribute to CP/CPPS pathogenesis. While these mechanisms have not yet been directly verified in CP/CPPS-specific contexts, their conservation across inflammatory diseases makes them promising hypotheses warranting future investigation.

### 5.1 Putative anti-inflammatory mechanisms

In a study of diabetic retinopathy, Zhou et al (34). investigated the role of bone marrow mesenchymal stem cell (BM-MSC)-derived exosomal miR-185-5p in regulating angiogenesis and inflammatory response. CXCL8 (IL-8) was identified as a direct target of miR-185-5p and was found to be highly expressed in high-glucose-treated human retinal microvascular endothelial cells. BM-MSC-derived exosomes inhibited cell proliferation, angiogenesis, and the production of inflammatory factors (VEGF, TNF-α, IL-1β, IL-6), whereas miR-185-5p inhibitor partially reversed these effects. Although this pathway has not been investigated in CP/CPPS, CXCL8/IL-8 is a well-established pro-inflammatory cytokine in CP/CPPS pathogenesis, and exosomal miRNAs targeting this pathway may represent a broader regulatory mechanism of inflammation. Whether miR-185-5p or functionally similar miRNAs regulate CXCL8/IL-8 expression in prostate cells warrants future investigation.

In another study on microscopic polyangiitis (MPA), a systemic vasculitis characterized by leukocyte infiltration, Zhu Y et al (35). identified that plasma exosomes from MPA patients were enriched in miR-1287-5p. Mechanistically, miR-1287-5p directly targets the E3 ubiquitin ligase CBL in human umbilical vein endothelial cells, leading to decreased CBL protein expression. This resulted in increased mRNA expression of inflammatory factors (IL-6, IL-8, MCP-1) and adhesion molecules (ICAM-1, E-selectin), and promoted neutrophil adhesion to endothelial cells. Conversely, miR-1287-5p inhibition reversed these effects. Although this pathway has not been investigated in CP/CPPS, the involvement of similar inflammatory mediators (IL-6, IL-8, MCP-1) in CP/CPPS pathology suggests that exosomal miRNA-mediated targeting of ubiquitin ligases or other regulatory proteins may represent a broader mechanism of inflammatory regulation. Future studies should explore whether CP/CPPS-derived exosomes carry miRNAs that target CBL or related ubiquitin ligases in prostate cells, and whether such mechanisms contribute to local inflammation.

### 5.2 Putative immune modulation mechanisms

In a study of colorectal cancer, Zhao Q et al (36). investigated the role of exosomal miR-191 in modulating macrophage polarization within the tumor microenvironment. The authors found that inhibition of miR-191 in colorectal cancer cells suppressed M2 polarization of macrophages. After coculture of macrophages, inhibition of miR-191 induced ROS production, ferroptosis, and apoptosis of cancer cells. Silencing of exosomal miR-191 from cancer cells prevented M2 polarization of macrophages and weakened tumor development by inducing ferroptosis. *In vivo* experiments further confirmed that exosomal miR-191 accelerated cancer progression in colorectal cancer nude mice by promoting M2 polarization of macrophages. Although this pathway has not been investigated in CP/CPPS, macrophage polarization—particularly the balance between pro-inflammatory M1 and anti-inflammatory M2 phenotypes—has been increasingly recognized as a critical determinant of inflammation resolution in CP/CPPS. The finding that exosomal miRNAs can modulate M2 macrophage polarization suggests that CP/CPPS-derived exosomal miRNAs might similarly influence macrophage phenotypes in the prostatic microenvironment. Future studies should investigate whether miR-191 or related miRNAs are present in CP/CPPS-derived exosomes and whether they contribute to M1/M2 imbalance and chronic inflammation persistence in CP/CPPS.

In a study of hepatocellular carcinoma, Yu H et al (37). investigated the role of tumor-derived exosomal miR-21-5p in mediating macrophage polarization. HCC cell-derived exosomes significantly promoted THP-1 macrophages to differentiate into M2-like macrophages, characterized by high production of TGF-β and IL-10. Bioinformatics analysis indicated that exosomal miR-21-5p was closely related to tumor-associated macrophage differentiation and associated with unfavorable prognosis. Mechanistically, miR-21-5p directly targeted RhoB 3’-UTR in THP-1 cells, and downregulated RhoB levels weakened MAPK signaling pathways. Notably, miR-21-5p has been identified as one of the most significantly upregulated miRNAs in EPS from CP/CPPS patients (21), and its levels correlate with symptom severity. However, whether CP/CPPS-derived exosomal miR-21-5p exerts similar effects on macrophage polarization in the prostatic microenvironment remains unknown. Given that miR-21-5p is elevated in CP/CPPS patients and that macrophage dysfunction is a feature of CP/CPPS pathology (22), we hypothesize that exosomal miR-21-5p may contribute to immune dysregulation by promoting M2-like polarization or inhibiting inflammatory macrophage responses, thereby perpetuating chronic inflammation.

### 5.3 Putative anti-oxidative stress mechanisms

In a study of hyperoxia-induced lung injury, Zou D et al (38). investigated whether melatonin-pretreated mesenchymal stem cell-derived exosomes (MT-Exo) carrying miR-18a-5p could alleviate oxidative stress and inflammatory injury. MT-Exo effectively inhibited hyperoxia-induced oxidative stress, inflammatory injury, and apoptosis in lung epithelial cells, while these effects were weakened by miR-18a-5p knockdown in MSCs. Mechanistically, miR-18a-5p reduced PUM2 expression in lung epithelial cells by directly targeting PUM2. PUM2 inactivated the Nrf2/HO-1 signaling pathway by promoting DUB3 mRNA decay post-transcriptionally. MT-Exo carrying miR-18a-5p reduced hyperoxia-mediated lung injury through activating the Nrf2/HO-1 pathway. Although this pathway has not been investigated in CP/CPPS, the Nrf2/HO-1 signaling pathway is a central regulator of antioxidant defense and has been implicated in CP/CPPS pathology (39). Given that oxidative stress is a well-established feature of CP/CPPS, and that exosomal miRNAs have been shown to modulate Nrf2 signaling in other disease model, we hypothesize that miR-18a-5p or functionally similar miRNAs may regulate the Nrf2/HO-1 axis in prostate cells, representing a potential therapeutic target for oxidative damage in CP/CPPS (40). Future studies should examine whether miR-18a-5p is present in CP/CPPS-derived exosomes and whether modulation of the PUM2/DUB3/Nrf2/HO-1 pathway can alleviate oxidative stress in the prostatic microenvironment.

### 5.4 Summary

**Figure 2:**
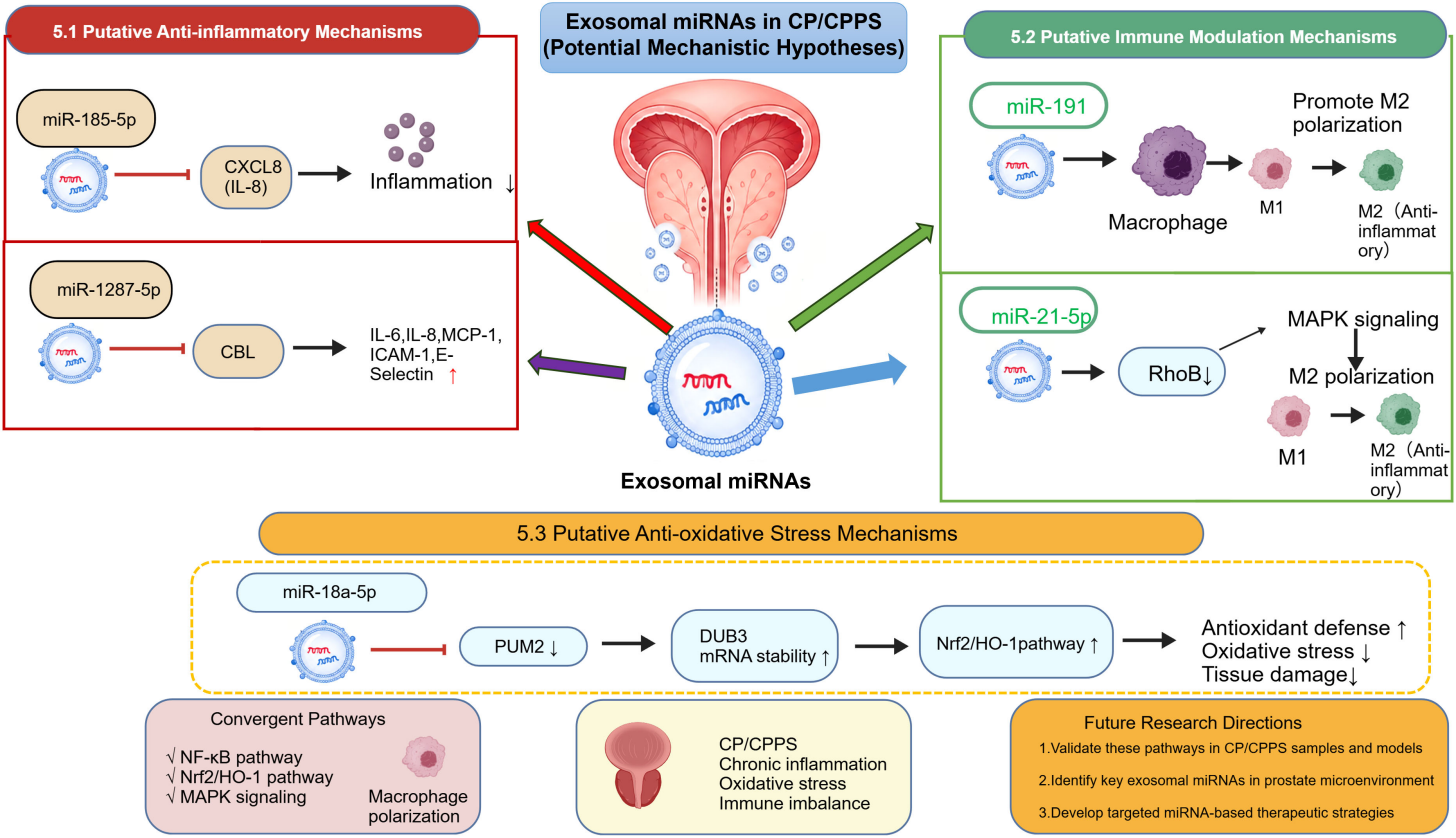
Hypothetical working model of potential exosomal miRNA-mediated mechanisms in CP/CPPS pathogenesis.

The mechanisms outlined above represent testable hypotheses rather than established pathogenic pathways in CP/CPPS. Across the three categories—anti-inflammatory, immune modulation, and anti-oxidative stress—several common themes emerge: (1) exosomal miRNAs frequently target key signaling nodes such as NF-κB, Nrf2/HO-1, and MAPK pathways; (2) macrophage polarization represents a recurring target of exosomal miRNA-mediated immune regulation; and (3) the convergence of multiple miRNAs on shared pathways suggests conserved mechanisms of inflammation and oxidative stress regulation across diseases. Notably, miR-21-5p is of particular interest to CP/CPPS research, as it is consistently elevated in EPS of CP/CPPS patients (21) and has been shown to promote M2 macrophage polarization in cancer models (37), raising the hypothesis that it may contribute to immune dysregulation in the prostatic microenvironment. Future studies should prioritize validation of these pathways in CP/CPPS patient samples and animal models to translate these hypotheses into clinical applications. The potential mechanism of exosomal miRNAs in chronic prostatitis is shown in **Table 3**. The hypothetical mechanisms outlined above are conceptually integrated in **Figure 2**, which provides a visual framework distinguishing these putative pathways from the directly validated networks summarized in **Figure 1**.

**Table 3:** The potential mechanism of exosomal miRNA in chronic prostatitis.

Mechanism category	miRNA	Key target/pathway	Validation status	Reference
Anti-inflammatory	miR-185-5p	CXCL8/IL-8	Inferred from diabetic retinopathy	(34)
Anti-inflammatory	miR-1287-5p	CBL → IL-6/IL-8/MCP-1↑	Inferred from microscopic polyangiitis	(35)
Immune modulation	miR-191	M2 macrophage polarization	Inferred from colorectal cancer	(36)
Immune modulation	miR-21-5p	RhoB → MAPK → M2 polarization	Inferred from hepatocellular carcinoma	(37)
Anti-oxidative stress	miR-18a-5p	PUM2 → DUB3 → Nrf2/HO-1	Inferred from hyperoxia-induced lung injury	(38)

## 6 Research progress on the clinical application of exosomal miRNAs in chronic prostatitis

The distinct expression profiles of exosomal miRNAs in EPS and urine from CP/CPPS patients, as detailed in Section 3, have positioned them as promising candidates for liquid biopsy-based diagnostics. Beyond diagnosis, exosomal miRNAs also hold potential as therapeutic targets and drug delivery vehicles. This section reviews the current state of clinical translation, highlighting both opportunities and challenges.

### 6.1 Diagnostic biomarkers

The differential expression of exosomal miRNAs in accessible biofluids—particularly EPS and urine—offers a non-invasive approach for CP/CPPS diagnosis. Chen et al. (21) employed a three-phase discovery-to-validation design and identified five miRNAs (miR-21-5p, miR-30a-5p, miR-30d-5p, miR-103a-3p, and miR-141-3p) that were significantly upregulated in EPS of CP/CPPS patients. Receiver operating characteristic (ROC) curve analysis demonstrated that miR-21-5p achieved the highest diagnostic accuracy, with an area under the curve (AUC) of 0.891, followed by miR-141-3p (AUC: 0.86) and miR-103a-3p (AUC: 0.84). In a longitudinal validation cohort, alleviation of pelvic pain symptoms was accompanied by significant downregulation of miR-21-5p, miR-103a-3p, and miR-141-3p, suggesting their dual utility as both diagnostic and prognostic biomarkers.

Ouyang et al. (22) identified a distinct panel of chronic pain-related miRNAs in EPS-derived extracellular vesicles, including miR-204-5p, let-7d-3p, let-7b-3p, let-7c-3p, miR-146a-5p, and miR-320a-5p. These miRNAs were differentially expressed between CP/CPPS patients and healthy controls and may serve as specific diagnostic biomarkers. However, quantitative performance metrics (AUC, sensitivity, specificity) were not reported in the original study, highlighting the need for further validation in diagnostic cohort studies.

Urine represents a readily accessible and completely non-invasive biofluid for diagnostic testing. Schneider et al. (27) identified a panel of eight prostate cancer-associated miRNAs—including hsa-miR-501, hsa-miR-20a, and hsa-miR-106—that were significantly upregulated in urinary exosomes from CP/CPPS patients. Functional assays revealed that these patient-derived urinary exosomes induced upregulation of proinflammatory genes (CCR2, TLR2) and proto-oncogenic transcription factors (MYB, JUNB) in THP-1 monocytes, maintaining them in an M0-like state and inhibiting M2 anti-inflammatory polarization. Notably, these miRNAs overlap with established prostate cancer biomarkers, suggesting that CP/CPPS may constitute a molecular risk state for prostate carcinogenesis. This finding has important clinical implications: CP/CPPS patients with long-standing disease may warrant more vigilant prostate cancer screening, and the presence of these miRNAs in CP/CPPS urine may confound urine-based cancer screening tests, necessitating separate reference ranges for patients with chronic inflammation.

Despite these promising findings, the translation of exosomal miRNA biomarkers into clinical practice faces several challenges. First, the lack of standardized protocols for exosome isolation, miRNA extraction, and data normalization contributes to poor reproducibility across studies. Second, the specificity of these miRNAs for CP/CPPS versus other urological conditions (e.g., benign prostatic hyperplasia, prostate cancer) requires further validation in large-scale, multi-center cohorts. Third, physiological variations and comorbid conditions may affect miRNA expression levels, necessitating robust reference ranges. Future diagnostic strategies should shift from the pursuit of a single “gold standard” biomarker toward the construction of diagnostic models based on multi-miRNA panels integrated with clinical parameters (e.g., NIH-CPSI scores).

### 6.2 Therapeutic targets

The causal role of specific miRNAs in CP/CPPS pathogenesis, established through gain-of-function and loss-of-function studies, positions them as attractive therapeutic targets. Song et al. (30) demonstrated that miR-203a-3p antagomir-loaded exosomes derived from prostate epithelial cells successfully targeted the prostate and alleviated prostatitis by inhibiting the DUSP5-ERK1/2 pathway in CP/CPPS-A model rats, providing proof-of-concept for miRNA-based intervention.

Lu et al. (31) showed that the traditional Chinese medicine Anemone tomentosa alleviated EAP-induced chronic inflammation by upregulating prostasome-derived miR-146a-5p, which targets TRAF6 and suppresses TLR-4/NF-κB pathway activation, providing molecular mechanism evidence for herbal therapy in CP/CPPS. In a separate study, Wang et al. (33) demonstrated that N-acetylcysteine (NAC) ameliorated carrageenan-induced prostatitis by restoring miR-141 expression, which targets Keap1 and activates Nrf2-mediated antioxidant responses.

Beyond small molecules and phytochemicals, engineered exosomes themselves offer a targeted delivery platform for miRNA therapeutics. Song et al. (30) constructed miR-203a-3p antagomir-loaded exosomes derived from prostate epithelial cells that demonstrated prostate-targeting capability and significantly reduced inflammatory cell infiltration and MCP-1 expression in prostate tissue. Peng et al. (32) showed that hiPSC-MSC-derived extracellular vesicles attenuated CP/CPPS in rats by immunoregulation, with significant reduction in NIH-CPSI-like scores and marked decrease in inflammatory cell infiltration.

However, the translation of miRNA-based therapeutics from bench to bedside faces substantial hurdles. The primary challenges lie in delivery, stability, and specificity. Naked miRNAs are rapidly degraded *in vivo*, necessitating safe and effective delivery vehicles. Moreover, given that a single miRNA can regulate multiple targets, achieving prostate-specific delivery while minimizing off-target effects in other organs is paramount (41). The blood-prostate barrier and cellular uptake efficiency further complicate targeted delivery. Future clinical strategies will therefore focus on developing next-generation formulations—such as chemically modified miRNAs (e.g., GalNAc-conjugated anti-miRs) or encapsulation in targeted nanoparticles—to enhance bioavailability and safety. The ultimate goal is to shift from broad systemic modulation toward precise, local manipulation of the prostatic miRNA network to restore inflammatory homeostasis.

### 6.3 Drug delivery systems

Beyond serving as therapeutic targets, exosomes themselves are being harnessed as nature-inspired drug delivery vehicles for treating CP/CPPS. Their inherent biocompatibility, low immunogenicity, and capacity to penetrate biological barriers render them superior to many synthetic nanocarriers (42).

Preliminary studies utilizing mesenchymal stem cell (MSC)-derived exosomes have demonstrated significant therapeutic efficacy in rat models of CP/CPPS. Peng et al. (32) demonstrated that hiPSC-MSC-derived extracellular vesicles significantly alleviated pain and inflammation in CP/CPPS rats by regulating Th cell imbalance. Administration of these vesicles resulted in a significant reduction in NIH-CPSI-like scores and a marked decrease in inflammatory cell infiltration and fibrosis in prostate tissue sections.

More recently, a novel engineering strategy has emerged to overcome the suboptimal targeting of natural exosomes. In a study on CP/CPPS treatment, ADSC-derived sEVs were surface-modified with CD86 antibodies using click chemistry, generating CD86-sEVs that specifically target M1 macrophages (43). These engineered sEVs significantly accelerated M1 macrophage polarization to the anti-inflammatory M2 phenotype and upregulated anti-inflammatory factors *in vitro*. *In vivo*, CD86-sEVs targeted the prostatic lesion region, alleviated chronic pelvic pain, and inhibited inflammation by promoting M1/M2 phenotype shift. miRNA array analysis identified specific miRNAs—miR-26a, miR-147, miR-17, miR-21, miR-182, and miR-451a—within CD86-sEVs that likely contributed to these observed effects (43).

However, the clinical translation of exosome-based therapeutics faces substantial manufacturing and regulatory hurdles. Key challenges include developing GMP-compliant production processes scalable for mass manufacture, establishing robust quality control metrics for heterogeneous exosome populations, and ensuring consistency in drug loading capacity and targeting efficiency (44). Future research should focus on engineering ‘customized’ exosomes via parental cell modification or post-isolation processing to enhance affinity for specific prostate cell types (e.g., epithelial or stromal cells). Such advancements would enable high-precision, cell-specific immunomodulation, offering a promising avenue for the treatment of CP/CPPS and related inflammatory disorders.

### 6.4 Summary of clinical translation

Exosomal miRNAs hold significant promise across the three pillars of clinical application—diagnosis, therapy, and drug delivery. In diagnostics, EPS- and urine-derived miRNAs offer non-invasive biomarkers with demonstrated correlation with symptom severity, though standardization and validation in large cohorts are urgently needed. In therapeutics, miRNA targeting has shown preclinical efficacy, yet delivery specificity and safety remain major barriers. In drug delivery, exosomes offer a biocompatible platform, with engineered surface modifications providing enhanced targeting capabilities. The integration of these three applications—using exosomal miRNA signatures to guide therapy and employing engineered exosomes for targeted delivery—represents a future paradigm for precision medicine in CP/CPPS. However, rigorous clinical trials are essential to establish safety and efficacy in human patients, and regulatory frameworks for exosome-based therapeutics must be developed to facilitate clinical translation. Clinical application summary is shown in **Table 4**.

**Table 4:** Clinical application summary table.

Application	Source	Key miRNAs/exosomes	Key findings	Reference
Diagnostic Biomarkers	EPS	miR-21-5p, miR-141-3p, miR-103a-3p	AUC: 0.891 (miR-21-5p); correlates with symptom severity	(21)
	EPS	miR-204-5p, let-7 family, miR-146a-5p, miR-320a-5p	Pain-related; potential specific biomarkers	(22)
	Urine	miR-501, miR-20a, miR-106	PCa-associated; risk stratification potential	(27)
Therapeutic Targets	Engineered exosomes	miR-203a-3p antagomir	Prostate targeting; alleviates prostatitis *in vivo*	(30)
	Herbal medicine	miR-146a-5p (Anemone tomentosa)	Upregulates miR-146a; suppresses TLR4/NF-κB	(31)
	Pharmacological	miR-141 (NAC)	Restores miR-141; activates Nrf2 antioxidant pathway	(33)
Drug Delivery	hiPSC-MSC-EVs	Immunoregulation	Reduces NIH-CPSI scores; decreases inflammation	(32)
	CD86-sEVs	miR-26a, -147, -17, -21, -182, -451a	M1→M2 macrophage polarization; targets prostate	(43)

## 7 Discussion

The present review synthesizes the current evidence on exosomal miRNAs in CP/CPPS, consolidating their role as pivotal orchestrators of cellular crosstalk within the local urogenital microenvironment. The differential expression profiles observed in patient biofluids—particularly EPS and urine—are not merely passive biomarkers but reflect an active, dynamic network of intercellular communication. As detailed in Section 4, exosomal miRNAs fuel a vicious cycle of pathogenesis by simultaneously propagating pro-inflammatory cascades, disabling anti-inflammatory brakes, inducing oxidative stress, and modulating immune cell function. Specifically, miR-155 and miR-203a-3p drive pro-inflammatory signaling through TLR4/NF-κB and ERK1/2/MCP-1 axes, respectively, while miR-146a-5p functions as a critical anti-inflammatory brake by targeting TRAF6 and suppressing TLR4/NF-κB activation (28–31). The miR-141/Keap1/Nrf2 pathway represents a directly validated antioxidant mechanism (33), and exosomal miRNAs from patient urine and engineered vesicles modulate monocyte and macrophage function (27, 32). Collectively, these findings support the concept of CP/CPPS as a complex ecosystem disorder perpetuated by continuous, miRNA-mediated pathological signaling between epithelial, stromal, and immune cells.

The mechanistic evidence discussed in this review is derived from various animal models, each with distinct pathological characteristics and limitations. The EAP model, induced by immunization with prostate antigens, closely mimics the autoimmune component of CP/CPPS and has been instrumental in elucidating T cell-mediated immune responses and the roles of miR-155 and miR-146a (29, 31). However, it primarily reflects autoimmune mechanisms and may not fully recapitulate the non-immune etiologies seen in many CP/CPPS patients. The carrageenan-induced CNP model induces sterile inflammation through direct chemical irritation, allowing for the study of acute-to-chronic inflammatory transitions and oxidative stress pathways, as exemplified by the miR-141/Keap1/Nrf2 findings (33). Yet it lacks the neuropathic pain and pelvic floor dysfunction components characteristic of human CP/CPPS. The hormone-induced model captures the impact of estrogen predominance on prostate inflammation and apoptosis (35), but is more reflective of aging-related changes than of the broader CP/CPPS population. Each model offers unique advantages for dissecting specific pathological pathways, yet no single model fully captures the multifactorial nature of human CP/CPPS. Therefore, conclusions drawn from any one model should be interpreted with caution, and findings should ideally be validated across multiple complementary models to establish robust and broadly applicable mechanisms.

Beyond model-specific considerations, several lines of evidence suggest a molecular overlap between CP/CPPS and prostate cancer (PCa). Schneider et al. (27) demonstrated that urinary exosomes from CP/CPPS patients carry PCa-specific miRNAs (e.g., miR-501, miR-20a, miR-106) that activate proto-oncogenic transcription factors in monocytes. Serum miRNA studies have further revealed that miR-223-3p and miR-223-5p are similarly downregulated in CP and PCa patients compared to BPH controls (45), suggesting that shared inflammatory pathways may drive miRNA alterations in both conditions. Clinically, this molecular overlap poses a twofold challenge: CP/CPPS patients with abnormal miRNA profiles may require more vigilant cancer screening, and the presence of CP/CPPS may confound miRNA-based PCa diagnostic tests, necessitating separate reference ranges for patients with chronic inflammation.

A critical caveat of this review is the reliance on evidence derived from non-CP/CPPS disease models to formulate mechanistic hypotheses (Section 5). While cross-disease extrapolation is a prevalent strategy in current CP/CPPS research—grounded in the evolutionary conservation of key signaling pathways (46)—it presents a significant translational gap. Many proposed mechanisms, such as miR-330-5p-mediated regulation of the NLRP3 inflammasome or miR-124-3p-driven antioxidant effects, lack direct experimental validation within the specific microenvironment of CP/CPPS. To address this, we have explicitly distinguished between mechanisms directly validated in CP/CPPS models (Section 4) and those inferred from other diseases (Section 5), framing the latter as testable hypotheses requiring CP/CPPS-specific verification.

Beyond mechanistic uncertainties, the field faces several structural limitations. Existing clinical studies are predominantly underpowered, with small sample sizes limiting statistical robustness; large-scale, multi-center cohorts are urgently needed. The absence of standardized protocols for exosome isolation and characterization introduces significant inter-study heterogeneity. Furthermore, current research has largely focused on isolated pathways or single miRNAs, lacking the systems-level integration necessary to capture the full complexity of the disease network. Future research must prioritize rigorous clinical trials, universal standardization protocols, and the development of engineered exosome-based delivery systems that overcome biological barriers—including the blood-prostate barrier—to achieve prostate-specific targeting. Leveraging cutting-edge technologies such as CRISPR-based gene editing, single-cell sequencing, and multi-omics integration will facilitate a granular understanding of exosomal miRNA networks. Ultimately, targeting the exosomal miRNA network offers a novel, precision medicine strategy to break the cycle of chronicity and restore homeostasis in patients with CP/CPPS.
